# Development of a quasi‐humanoid phantom to perform dosimetric and radiobiological measurements for out‐of‐field doses from external beam radiation therapy

**DOI:** 10.1002/acm2.13514

**Published:** 2022-02-01

**Authors:** Marta Kruszyna‐Mochalska, Agnieszka Skrobala, Piotr Romanski, Adam Ryczkowski, Wiktoria Suchorska, Katarzyna Kulcenty, Igor Piotrowski, Dorota Borowicz, Natalia Matuszak, Julian Malicki

**Affiliations:** ^1^ Electroradiology Department University of Medical Sciences Poznan Poland; ^2^ Medical Physics Department Greater Poland Cancer Centre Poznan Poland; ^3^ Radiobiology Laboratories Medical Physics Department Greater Poland Cancer Centre Poznan Poland

**Keywords:** humanoid phantoms, out‐of‐field doses, radiobiological response, risk modeling

## Abstract

Our understanding of low dose, out‐of‐field radiation and their radiobiological effects are limited, in part due to the rapid technological advances in external beam radiotherapy, especially for non‐coplanar and dynamic techniques. Reliable comparisons of out‐of‐field doses produced by advanced radiotherapy techniques are difficult due to the limitations of commercially available phantoms. There is a clear need for a functional phantom to accurately measure the dosimetric and radiobiological characteristics of out‐of‐field doses, which would in turn allow clinicians and medical physicists to optimize treatment parameters. We designed, manufactured, and tested the performance of a quasi‐humanoid (Q‐H) adult phantom. To test the physics parameters, we used computed tomography (CT) scans of assembled Q‐H phantom. Static open field and dynamic techniques were measured both in‐ and out‐of‐field with ionization chambers and radiochromic films for two configurations (full solid and with water‐filled containers). In the areas simulating soft tissues, lung, and bones, median Hounsfield units and densities were, respectively: 129.8, ‐738.7, 920.8 HU and 1.110, 0.215, 1.669 g/cm^3^. Comparison of the measured to treatment planning systems (TPS) in‐field dose values for the sample volumetric arc therapy (VMAT) (6 MV flattening filter‐free (FFF)) plan, 96.4% of analyzed points passed the gamma evaluation criteria (L2%/2 mm, threshold (TH) 10%) and less than 1.50% for point dose verification. In the two phantom configurations: full poly(methyl) methacrylate (PMMA) and with water container, the off‐axis median doses for open field, relative to the central axis of the beam (CAX) were similar, respectively: 0.900% versus 0.907% (15 cm distance to CAX); 0.096% versus 0.120% (35 cm); 0.018% versus 0.018% (52 cm); 0.009% versus 0.008% (74 cm). For VMAT 6 MV FFF, doses relative the CAX were, respectively: 0.667% (15 cm), 0.062% (35 cm), 0.019% (52 cm), 0.016% (74 cm). The Q‐H phantom meets the International Commission on Radiation Units and Measurements (ICRU) and American Association of Physicists in Medicine (AAPM) recommended phantom criteria, providing medical physicists with a reliable, comprehensive system to perform dose calculation and measurements and to assess the impact on radiobiological response and on the risk of secondary tumor induction.

## 1 INTRODUCTION

In clinical radiotherapy, out‐of‐field radiation doses could negatively impact the patients. Consequently, it is essential to determine the out‐of‐field radiation doses (both close to and far from the field edge) and their characteristics, including the following: dosimetric measurements of low dose radiation,^1^ dose distribution and energy spectrum (calculated by advanced modeling methods such as Monte Carlo^2^, ^3^), and the impact on radiobiological response^4^, ^5^ to determine the risk of inducing a secondary tumor.^6^, ^7^

To fully understand the impact of dynamic radiotherapy techniques such as volumetric arc therapy (VMAT) or tomotherapy,^8^ as well as non‐coplanar techniques such as CyberKnife and stereotactic body radiation therapy (SBRT), it is essential to assess out‐of‐field doses using a consistent and repeatable process. In other words, different treatment plans and techniques should be subjected to the same study conditions to obtain precise dose and energy distributions for comparison. Comparable conditions are essential to determine the true correlation between the dose and radiobiological response, which in turn allows clinicians to estimate individual response to reduce exposure to low doses when necessary. Achieving consistent, uniform measurements requires a functional phantom and appropriate measurement methods (both dosimetric and radiobiological). However, the currently available phantoms, including anthropomorphic phantoms (e.g., Alderson‐Rando phantom),^9^ water or slab phantoms,^10^ or combined phantoms,^11^ generally provide only a limited number of points where different types of detectors and flasks containing cell lines can be inserted.^12^ Moreover, the condition in water and slab phantoms are not sufficiently similar to those prevailing in the human body.

Although there is a large published body of evidence on low dose radiation, it is difficult to directly compare the results obtained in those studies due to the wide inter‐study heterogeneity in terms of aims, methods, and phantom types, among other factors. This is a well‐known and widely recognized issue, as indicated in the recently released American Association of Physicists in Medicine (AAPM) 158 report,^13^ which emphasized the importance of evaluating out‐of‐field doses in order to estimate the risk of inducing secondary tumors in patients treated with external beam radiotherapy (EBRT). AAPM 158 discusses the key aspects needed to reliably estimate the effects of low doses, one of which is the proper selection of the phantom. As that report noted, all of the currently available commercial phantoms present important limitations. Therefore, our group sought to design a phantom to overcome those disadvantages and other issues (e.g., pacemakers and other electronic devices, fetal dose, cardiac toxicity, skin dose) raised in the AAPM 158 report.

In this context, the objective of the present study was to design, manufacture, and evaluate a quasi‐humanoid (Q‐H) adult phantom. The phantom was specifically designed to enable the determination of the radiobiological response and dosimetric characteristics of out‐of‐field radiation from photon beam EBRT.

## 2 METHODS

This research work was carried out to design a novel Q‐H phantom that would allow for the following: (1) simultaneous assessment of dose distribution and radiobiological response; (2) measurement of both in‐ and out‐of‐field doses; (3) ability to use different dosimeter types (both passive and active) placed in relevant locations. Other essential characteristics of the phantom were as follows: similarity in size and shape to the human body; built‐in inhomogeneities with an appropriate electron density to ensure scatter and attenuation similar to the human body; universal design (to simulate arrangements, shapes, and sizes for different case studies); ease of assembly and positioning; high degree of reproducibility; and cost‐effectiveness.

**FIGURE 1:**
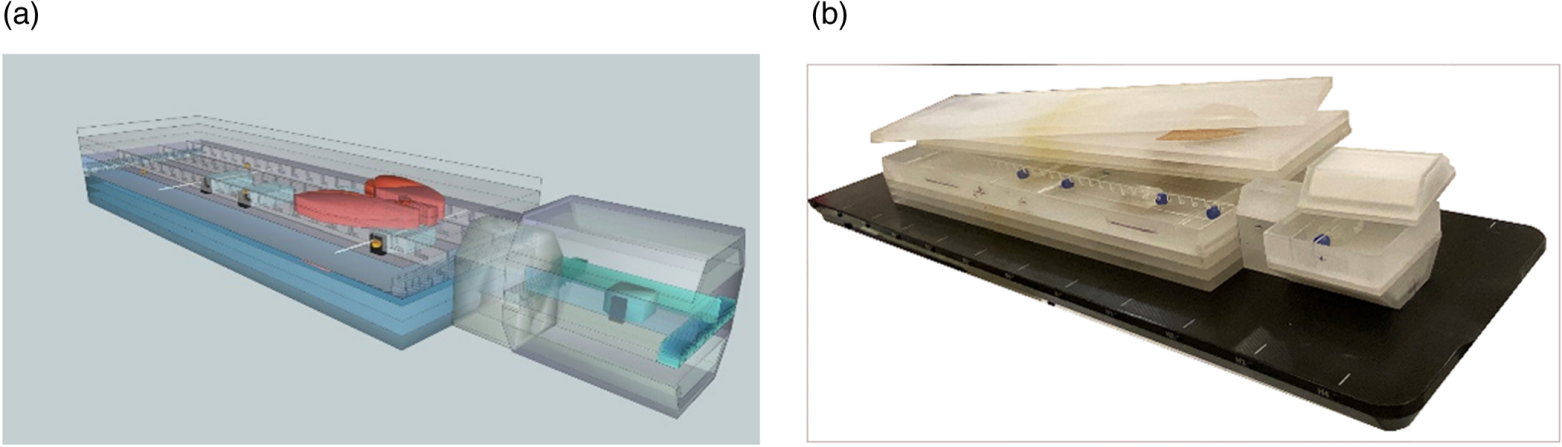
Design (a) and implementation (b) of quasi‐humanoid (Q‐H) phantom for dosimetric and radiobiological measurements. The phantom consists of slices with inhomogeneities simulating those present in human tissues, with gold fiducial markers for setup and inserts (containers) to irradiate cell flasks

Prior to beginning the design process, we reviewed the published literature on phantoms for non‐target doses (i.e., out‐of‐field doses, peripheral doses) for EBRT. The findings of that review of available phantom solutions are summarized in Table 1, which groups the phantoms in terms of their applications in measuring and assessing radiobiological response. Next, we prepared a list of required elements for the construction of a Q‐H phantom that would provide for optimal out‐of‐field dose testing as well as assessment of the impact of out‐of‐field radiation doses on cellular response. The phantom was designed (Figure 1a) and manufactured (Figure 1b) according to recommendations provided in International Commission on Radiation Units and Measurements (ICRU) 44^9^ and AAPM TG 158,^13^

**TABLE 1:** Characteristics of phantoms commonly used to measure out‐of‐field doses and radiobiological response versus the universal quasi‐humanoid (Q‐H) phantom

	Descriptions	Assumptions			
Type	Phantom material/size/elements	Measure point and type of detectors	Radiobiology response	Repositioning/RT techniques/cost	References
Simple phantom	Water scanning tanks/one size, different water levels/one element	To perform many scans, in various planes for a variety of detectors (most often waterproof active detectors) along and across radiation beams for static/gantry fixed fields	Great freedom for positioning (limitation only at the phantom edges), special inserts and fixation required	Large sizes limit freedom of positioning/only gantry fixed techniques/low cost—usually equipped in the department, possible costs related to inserts and immobilization	^13^, ^14^, ^15^
	Water filled/different sizes available/one element	Special guides and fixation/holder required for positioning detectors (waterproof or required inserts)	Great freedom for positioning (limitation only at the phantom edges), special inserts and fixation required	Detector positioning system required/possible advanced techniques/low cost	^1^, ^2^, ^5^, ^16^
	Solid phantoms/different size/many elements/enabled shape and size matching to ensure approximate dispersion in the patient's body	Limitation on the location of the detectors at any point, the need for special inserts for detectors/passive and some active	Requires significant reconstruction and special inserts	Repeatability issues in arranging the constructed structure/advanced techniques possible/low cost—usually equipped in the department, possible cost related to inserts and immobilization	^10^, ^11^, ^12^, ^17^, ^18^
Complex phantom	Anthropomorphic (adult or children)/one size/many elements/complex shape and contours of human anatomy with heterogeneities such as lungs and bony anatomy	Limitation on the location of the detectors at any point, usually passive (Gafchromic, TLD), use of other methods requires significant reconstruction	Requires significant reconstruction and special inserts	Repeatable positioning (IGRT system)/advanced techniques possible/high cost	^13^, ^19^, ^20^
	Q‐H phantom/different size/many elements/PMMA with elements of bone, lung and soft tissues, and water/main body and head and neck	Large degree of freedom in positioning (limited to 1 cm along and in height and 2.5 cm across of the active detectors in the inserts—part of the slab phantom)/most passive and active	Wide degree of freedom for positioning in a water container (limitations only at the phantom edges)	Repeatable positioning (IGRT system)/advanced techniques possible/low cost	Present study (Q‐H phantom)

Abbreviations: IGRT, image‐guided radiotherapy; PMMA, poly(methyl) methacrylate; RT, radiotherapy; TLD, thermoluminescent dosimeters.

**FIGURE 2:**
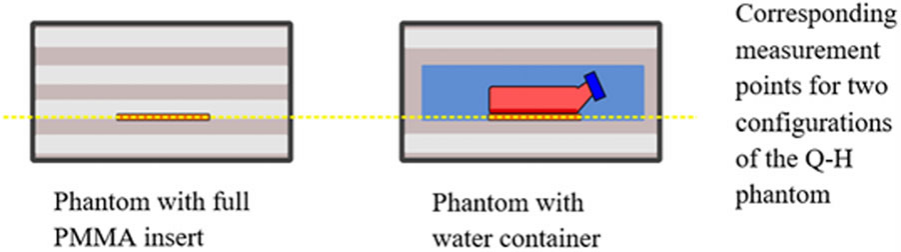
The out‐of‐field dose measured with dosimetric films (EBT3) in both configurations of phantom: full poly(methyl) methacrylate (PMMA) inserts and during simultaneous irradiation of the flasks in the water container.

The phantom was primarily constructed of poly(methyl) methacrylate (PMMA) combined with natural cork and gypsum to create inhomogeneities. After manufacturing the individual elements, a prototype phantom was built (Figure 1b). To test the functionality of the phantom, we assembled it into two different configurations by replacing one part with the appropriate inserts (equivalent in size), as follows (Figure 2): a PMMA layer with inserts for dosimetric measurements and a container with water and flask's stabilizers to assess radiobiological response. To check the reliability and construction of the Q‐H phantom, the in‐ and out‐of‐field dose measurement points were chosen at the same position in the phantom (corresponding positions) in both configurations.

Tomographic scans of the assembled Q‐H phantom were performed using Siemens SOMATOM Definition AS (120 kV X‐ray tube voltage, 1 mm slice thickness). The samples were taken with the Eclipse planning system (ARIA 15.6, Varian Medical Systems, USA). We then evaluated the physics parameters in the phantom, including the following: density, Hounsfield units (HU; range and median values), relative electron density (RED) for materials representing soft tissue, lung tissue, bone, and structural homogeneity.

To verify the adequacy of the newly built phantom, radiation dose measurements were performed for open fields for different energies. Based on created contours of chosen volumes, a sample treatment plan for the pelvic area was created using a dynamic technique (VMAT, 6 MV flattening filter‐free (FFF), SBRT protocol), which was delivered by linear accelerator (TrueBeam accelerator, Varian Medical Systems) for two conditions: (A) measurement of the in‐field dose distribution (prescribed target dose: 20 Gy) using dosimetric films Gafchromic EBT‐XD (Ashland, Bridgewater, NJ, USA) and an ionization chamber (Semiflex 0.125cc, PTW Freiburg), and (B) out‐of‐field dose distribution measurements (open field, dose in central axis of the beam (CAX): 532 Gy) measured with dosimetric films Gafchromic EBT3 (Ashland, Bridgewater, NJ, USA) in the both configurations of phantom: during simultaneous irradiation of the flasks in the water container and PMMA inserts (Figure 2). Measurements were also made for an exemplary dynamic treatment plan with an appropriately scaled dose (22 fraction of VMAT, 10 Gy per fraction, 6 MV FFF) for a phantom with a water container.

EBT‐XD and EBT3 radiochromic films (Ashland Inc., USA) were used to measure in‐field (20 Gy) and out‐of‐field doses (several cGy), respectively. The films were scanned on an Epson scanner with a resolution of 72 dpi in a 48‐bit format and analyzed using Verisoft software (PTW Freiburg) and ImageJ software (National Institutes of Health, Bethesda, MD, USA). The preparation, exposure, and analysis of the film detectors was carried out in accordance with the AAPM TG 235 report.^21^

## 3 RESULTS AND DISCUSSION

As noted in Section 2, prior to designing the phantom, we reviewed the literature on phantoms commonly used to measure non‐target doses. Table 1 summarizes the various phantoms used in low dose studies, general assumptions, and important limitations.

Our Q‐H phantom is comprised of three parts: main body, neck, and head. Each component consists of slices in the coronal plane, which allows researchers to use any measuring system, up to the maximum size for each body part (300 × 1000 × 500 mm^3^) and at the head (200 × 200 × 250 mm^3^) (Table 2, Figure 1). The essential features of the Q‐H phantom are summarized in Table 1, and its elements are described in Table 2.

**TABLE 2:** Description of the phantom elements designed to measure non‐target doses (width × length × height)

Anthropomorphic region	Main elements/material and heterogeneities (a)	Positioning system (b)	Additional elements for dosimetric measurements (c)	Additional elements for dosimetric measurements and radiobiological response (d)
Head	PMMA slices in the coronal plane: ‐200 × 200 × 20 mm^3^ (10×); ‐200 × 200 × 10 mm^3^ (5×); ‐bottom and top of the head—elements with isosceles trapezium base with the bone structure (plaster) thick 10 mm in the bottom and top part of the head.	‐4 cylindrical golden markers with 2 mm length and 1 mm diameter; ‐20 pieces pins for immobilization with 5 mm diameter and 10 mm length.	PMMA elements: ‐200 × 50 × 20 mm^3^ (3×); ‐200 × 20 × 20 mm^3^ (4×); ‐200 × 10 × 20 mm^3^ (2×); ‐50 × 20 × 20 mm^3^ (3×); ‐50 × 20 × 20 mm^3^ (3×) with central drill hole with 8 mm diameter along the entire length (cable grommet function); ‐50 × 20 × 20 mm^3^ with central drill hole with 25 mm length and 4 mm (2×) and 8 mm diameter (2×).	Container filled with water 200 × 200 × 50 mm^3^: ‐190 × 5 × 40 mm^3^ (4×) crossbars were preventing the ascent and movement of flasks, enabling irradiation of flasks with biological material anywhere in the container.
Body	PMMA slices in the coronal plane: ‐300 × 1000 × 20 mm^3^ (15×); ‐300 × 1000 × 10 mm^3^ (10×); ‐300 × 1000 × 20 mm^3^ (4×) with build‐in the material simulating of lung tissues (natural cork).	‐8 cylindrical golden markers with 2 mm length and 1 mm diameter; ‐50 pins with 5 mm diameter and 10 mm length that the layers do not move relative to each other.	PMMA elements: ‐300 × 200 × 20 mm^3^ (4×); ‐300 × 140 × 20 mm^3^ (4×); ‐300 × 100 × 20 mm^3^ (2×); ‐300 × 50 × 20 mm^3^ (2×); ‐300 × 20 × 20 mm^3^ (4×); ‐300 × 10 × 20 mm^3^ (2×); ‐100 × 20 × 20 mm^3^ (3×); ‐100 × 20 × 20 mm^3^ (3×) with central drill holes with 8 mm diameter along the entire length (cable grommet function); ‐100 × 20 × 20 mm^3^ with central drill holes with length 50 mm and a diameter 4 mm (2×) and diameter 8 mm (3×).	Container filled with water 300 × 1000 × 50 mm^3^: ‐290 × 5 × 5 mm^3^ (15×) rods—crossbars preventing the ascent and movement of flasks); ‐5 × 990 × 40 mm^3^ (7×) stabilizing bars with attachment hooks enabling simultaneous irradiation of multiple flasks with biological material anywhere in the container.
Neck	Bottom of the neck—100 × 100 × 100 mm^3^, with the cylindrical bone structure with 20 mm diameter and 100 mm length; top of the neck—element with isosceles trapezium base. The possibility of measuring and testing the radiobiological response was not provided.			

*Note*: These include functional layers (a) with inhomogeneities (materials simulating lung and bone tissue), (b) layers with embedded gold fiducial markers for positioning with image‐guided radiotherapy (IGRT), (c) layers consisting of inserts for different detectors with spacers to ensure uniform radiation dispersion, and (d) water containers, together with appropriate stabilizers, to permit the precise arrangement of containers with biological material.

Abbreviation: PMMA, poly(methyl) methacrylate.

The phantom is also equipped with several functional slides (Table 2), which can be used interchangeably depending on specific needs and applications. To evaluate the effect of out‐of‐field radiation, the Q‐H phantom with two insert configurations (dosimetric measurements and radiobiological response) was assembled.

**FIGURE 3:**
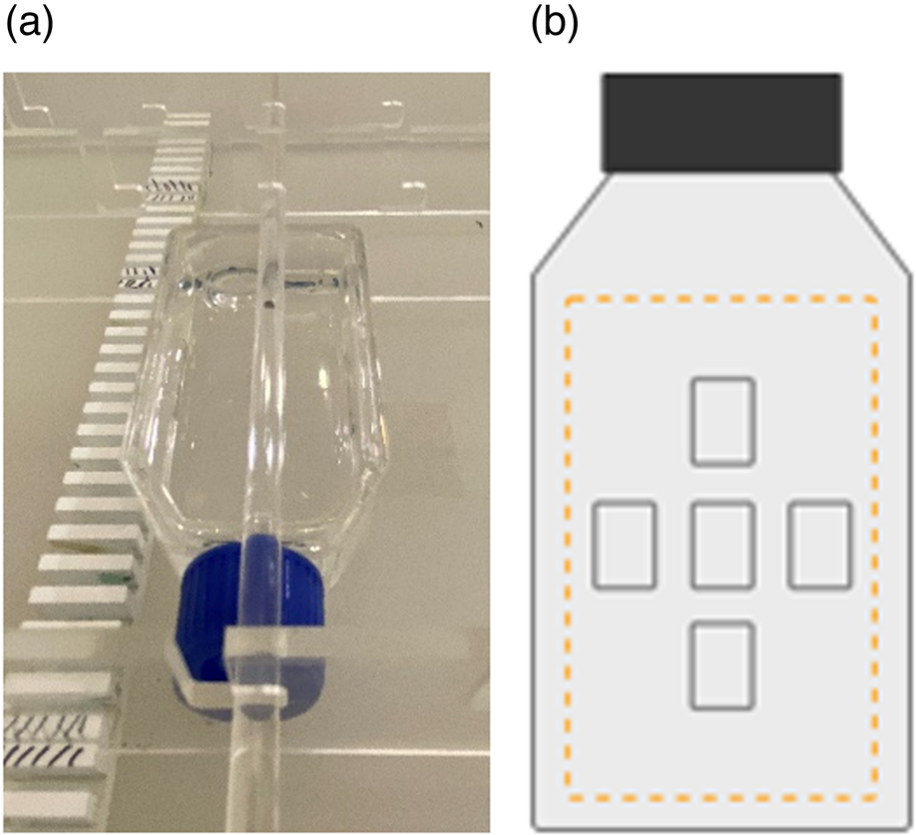
Water‐filled containers with numerous spacers and stabilizers to permit irradiation of cell flasks/detectors in any required position. The stabilizing bars with attachment hooks enable simultaneous irradiation of multiple flasks containing biological material anywhere in the container. The crossbars prevent the flasks from moving (a). The scheme of flask with simultaneous control through dosimetric films (orange line) with five regions of interest (ROIs), placed under the bottle (the distance between the film and cells is approximately 1 mm) (b)

In the first configuration, we used a container with water for irradiating biological material and with additional control of the in‐ and out‐of‐field absorbed doses. Two different sized water‐filled containers can be used in the head and body of the phantom. The availability of spacers and stabilizers along the entire length of the containers (Figure 3a) allows for detectors and/or flasks containing biological materials to be positioned anywhere inside the phantom to assess radiobiological response. Figure 3b shows the most common flask irradiation technique that we used, with simultaneous control of the cells within the layer (bottom of the bottle) through dosimetric films placed under the bottle (distance between the film and cells = approximately 1 mm). In order to prevent and eliminate air cavities we used cone beam computed tomography (CBCT) imaging after water filling.

In the second configuration, a layer of PMMA plates (equivalent in size to the container) were replaced with inserts and detectors at the same points as the films under the flasks in the first configuration to measure the radiation properties. The insert system for dosimetry measurements consists of elements that contain drill holes of various sizes to accommodate the range of available detectors (from 4 to 8 mm), and components to enable cabling to the detectors (cable grommets), and full elements to ensure scatter. A wide variety of detectors can be used, including: dosimetric films, ionization chambers, semiconductor detectors, and both passive and active dosimeters (all of which are commonly used to measure non‐target doses^13^). The phantom provides a large degree of freedom in positioning the detectors in the phantom slab (for active detectors limited to 1 cm in length and height and 2.5 cm in width for active detectors in the inserts), and/or in water‐filled containers (passive detectors: Gafchromic, thermoluminescent dosimeters (TLD)). In solid parts, the film dosimeters can be placed in the coronal plane only. If water container part is used it allows on measurements in all planes. The phantom enables dose measurements on the surface (e.g., using film or metal oxide semiconductor field effect transistor (MOSFET) detectors).

**FIGURE 4:**
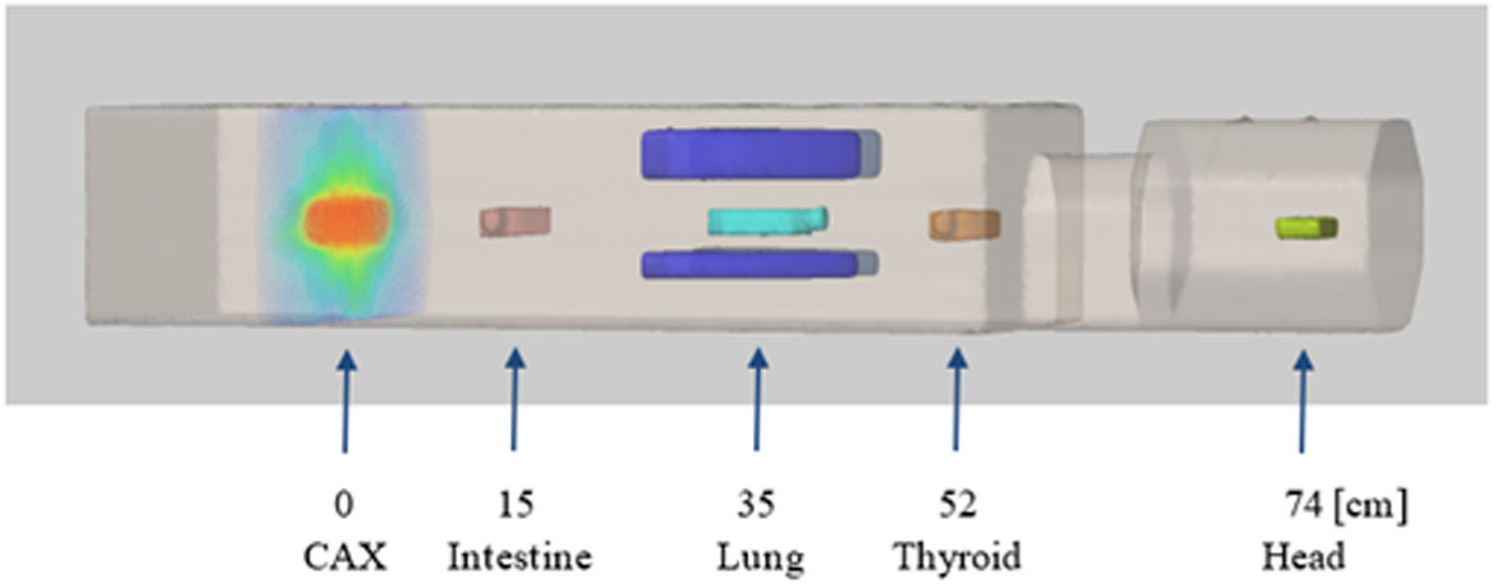
Illustration of quasi‐humanoid (Q‐H) phantom used to determine the effects of low out‐of‐field doses. The image shows a 3D visualization based on computed tomography (CT) scans, with an example of dose distribution for pelvic irradiation and selected measurement points simulating the localizations of organs in the Q‐H phantom

The phantom includes gold fiducial markers for setup to enable positioning using image‐guided radiotherapy (IGRT) systems (kV‐kV or kilovoltage CBCT (kV‐CBCT)/megavoltage CT (MVCT)). This ensures the repeatability of a given setup and allows us to determine the field boundary (and its reproducibility) to measure the distance to individual assessment points.^13^ Figure 4 illustrates a 3D visualization of the assembled Q‐A phantom based on CT scans, with an example of dose distribution for pelvic irradiation.

Physics parameters (density, HUs, and RED) were checked using the Eclipse planning system for the Q‐H phantom—which includes materials (PMMA, cork, and gypsum) designed to simulate soft tissues, bone, and lung tissues (Table 3).

**TABLE 3:** Physics parameters, including Hounsfield units (HU), density, and relative electron density (RED) in the quasi‐humanoid (Q‐H) phantom based on computed tomography (CT) scans of the phantom materials (to simulate soft tissue, bone, and lung tissues)

		HU				
Phantom	Tissue types	MIN	MAX	MEDIAN	Mass density (g/cm^3^)	RED
Q‐H phantom	Lung	‐799.0	‐688.4	‐738.7	0.215	0.202
	Bone	898.2	941.2	920.8	1.669	1.563
	Soft	118.7	139.3	129.8	1.110	1.074

In non‐target dose studies, treatment planning systems (TPS) used to calculate the distribution of doses at significant distances from the irradiated target are of limited utility. The energy spectrum outside the treatment field is lower than the in‐field spectrum, ranging from 200 to 300 keV for a nominal energy of 6 MV.^12^ In building an optimal phantom, it is essential that the individual components be constructed of materials whose characteristics (homogeneity, density, and effective atomic number) are well‐understood regard to. This is important to effectively and quickly define the Monte Carlo simulation parameters, calculate equivalent distances between the analyzed points, and to assess differences in terms of irradiation interactions with the matter. PMMA is commonly used to create phantoms, with an effective *Z* (Zeff) value of 6.56 (comparable to soft tissues, 7.64).^22^ According to ICRU 44 for PMMA, the ratio of photon interaction coefficients (*μ*/ρ)—the energy intervals within which the percentage difference between substitute and the reference tissue is less than 3% for energy range 0.15–50.00 MeV, and for differences ranging from 3% and 10%, the energy interval is 0.05–0.10 MeV.^9^

We validated the phantom by comparing measured dose values (using a semiflex 0.125 cc ionization chamber with correction for output) to TPS values, thereby obtaining the percent differences for the open field (10 × 10 cm^2^) at different energy levels, with a repeatability of <0.10%, as follows: 0.089% (6 MV); ‐0.146% (6 MV FFF); 0.145% (10 MV FFF); and ‐0.696% (15 MV).

**FIGURE 5:**
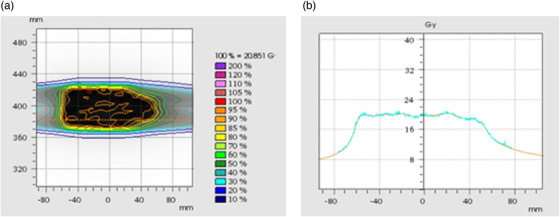
An example of the dose distribution planned for the volumetric arc therapy (VMAT) (6 MV flattening filter‐free (FFF)) technique plan for the quasi‐humanoid (Q‐H) phantom in the coronal plane (a) with a comparison of dose profiles measured using Gafchromic EBT‐XD films and the planned distribution in the field (b)

Verification of the sample VMAT (6 MV FFF) plan for irradiation of the prostate simulation area yielded the following results: for the ionization chamber placed at target center‐of‐mass, the differences were below 1.50%; for the plane verification (EBT‐XD dosimetric film) in the coronal slice, 96.4% of analyzed points passed the gamma evaluation criteria: L2%/2 mm with threshold (TH) 10%. Figure 5 shows an example of the planned dose distribution compared to the planned and measured profile functions.

**FIGURE 6:**
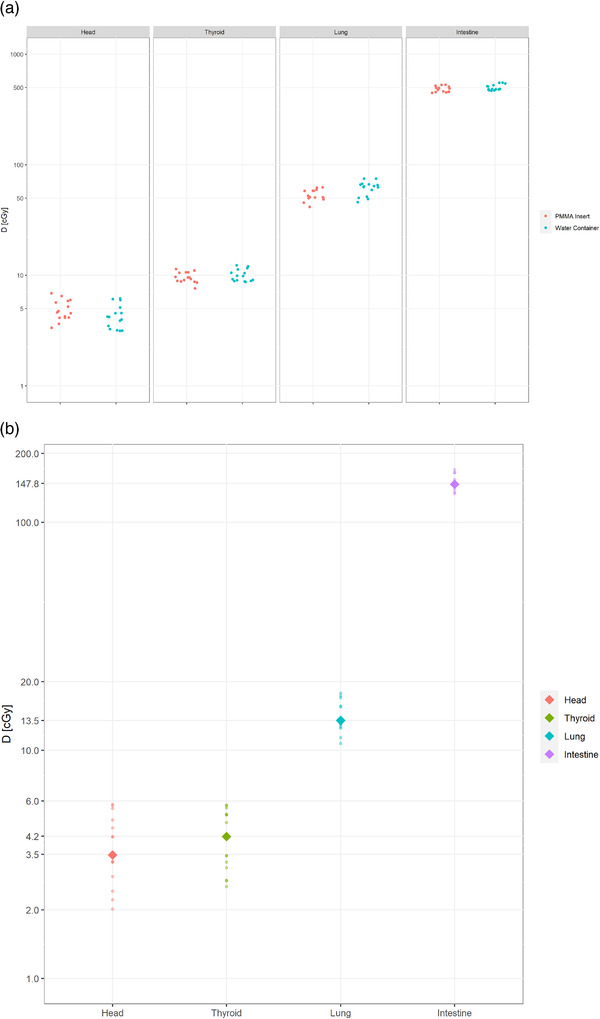
An example of the out‐of‐field dose measured (EBT3 film) at distances ≥15 cm from the central axis of the beam (CAX) for the open field (532 Gy to CAX, 6 MV flattening filter‐free (FFF)) for two configurations: poly(methyl) methacrylate (PMMA) insert and water container (a). Measurements for an exemplary dynamic treatment plan with an appropriately scaled dose (22 fractions of volumetric arc therapy (VMAT), 10 Gy per fraction, 6 MV FFF) for a phantom with a water container (b)

To compare the out‐of‐field doses for the two configurations of the Q‐H phantom which differed in terms of the elements included: one phantom had a water container for simultaneous irradiation of flasks and the second had full PMMA slices measurement at corresponding points (doses measured with EBT3 for five regions of interest (ROI) and three repetitions; Figures 3 and 6). The out‐of‐field doses were measured in the Q‐H phantom for commonly used 6 and 10 MV FFF photons at open field and for VMAT, in both configurations at the selected points at distance of 15, 35, 52, and 74 cm from the CAX (simulating of organs in the Q‐H phantom; Figure 4) using Gafchromic (EBT3) films according to scheme presented in Figure 2.

In this universal Q‐H phantom, dose distributions and the radiobiological response can both be determined simultaneously or separately in different configurations. In addition, tests may be performed at the corresponding points for a more detailed determination of the physical parameters. The off‐axis doses in the examined locations (corresponding points for two configurations presented in Figure 2) reached the level in the PMMA and water arrangements, respectively: 0.900% versus 0.907% (15 cm distance to CAX); 0.096% versus 0.120% (35 cm); 0.018% versus 0.018% (52 cm); 0.009% versus 0.008% (74 cm), relatively to the dose in CAX (Figure 6a). For VMAT 6 MV FFF plan we obtained, respectively: 0.667% (15 cm), 0.062% (35 cm), 0.019% (52 cm), 0.016% (74 cm). We recommend using simultaneous film dosimetry control to assess biological response, as the water level and irradiation condition of the flasks yield less reliable results than those achieved in solid phantoms at the same points.

The Q‐A phantom described in this paper has enormous potential for evaluating out‐of‐field radiation. The phantom can be used for radiation dose and radiobiological response measurements in most clinically relevant locations. It also allows for detailed testing at various points to comprehensively assess the physics parameters at those locations. The Q‐H phantom is also highly flexible, allowing for dose assessment and radiobiological response, both inside and outside the treatment field.

Another advantage of this novel Q‐H phantom is that it includes a tool to measure dose distributions for patients wearing a cardiac implantable electronic device (CIED) and pregnant women. In other words, it offers the ability to verify non‐target doses for individual patients, which is also recommended in AAPM 158 reports.^13^ This phantom could be adapted for other uses or studies through 3D printing or moving parts (in the water container) for techniques with motion tracking. In addition to its use in radiotherapy, it can also be used, when suitably adapted, in interventional radiology or nuclear medicine.

The extensive validation of the Q‐H phantom for out‐of‐field conditions was done for 6 and 10 MV FFF open field and for VMAT technique. The further study is required to cover other beams and techniques used in radiotherapy, as well as measure doses in other locations in the phantom than those tested.

## 4 CONCLUSION

The Q‐H phantom meets both ICRU and AAPM recommended criteria. The measurements made in this study confirm its value for the study of radiobiological and dosimetric response for out‐of‐field radiation from photon beam EBRT.

The Q‐H phantom described in this paper presents many solutions (simultaneous assessment of dose distribution and radiobiological response; measurement of both in‐ and out‐of‐field doses; ability to use different dosimeter types; etc.) and methods to offer researchers a tool to assess the dosimetry and radiobiological effects of non‐target radiation. The phantom provides a comprehensive system that enables measurements, as well as assessment of the impact of these doses on radiobiological response and on the risk of inducing a secondary tumor.

## AUTHOR CONTRIBUTIONS

*Conceptualization*: Marta Kruszyna‐Mochalska, Agnieszka Skrobala, and Julian Malicki. *Methodology*: Marta Kruszyna‐Mochalska, Adam Ryczkowski, Agnieszka Skrobala, Wiktoria Suchorska, Katarzyna Kulcenty, Igor Piotrowski, Natalia Matuszak, and Julian Malicki. *Measurements and analysis*: Marta Kruszyna‐Mochalska, Piotr Romanski, Adam Ryczkowski, and Dorota Borowicz. *Discussion and conclusions*: Marta Kruszyna‐Mochalska, Adam Ryczkowski, Igor Piotrowski, and Agnieszka Skrobala. *Writing—draft preparation*: Marta Kruszyna‐Mochalska, Agnieszka Skrobala, Piotr Romanski, and Julian Malicki. All authors have offered a substantial contribution to conception, design, analysis, interpretation, and approval of the manuscript. They reviewed and accepted the final version of the manuscript.

## CONFLICT OF INTEREST

The authors declare no conflict of interest.

## Data Availability

The data that support the findings of this study are available from the corresponding author upon reasonable request.
